# A qualitative study assessing cardiovascular risk factors: the accumulative stressors influencing societal integration of teenage African immigrants

**DOI:** 10.1186/s12889-015-2122-6

**Published:** 2015-08-15

**Authors:** Cheryl Zlotnick, Hadass Goldblatt, Efrat Shadmi, Daphna Birenbaum-Carmeli, Omer Taychaw

**Affiliations:** Cheryl Spencer Department of Nursing, University of Haifa, 199 Abba Khushi Avenue Mt Carmel, Haifa, Israel

**Keywords:** Adolescents, Cardiovascular risk, Stress, Immigrants, Discrimination

## Abstract

**Background:**

This study examines the nature of disparities in cardiovascular risk by exploring chronic stressors and other cardiovascular risk factors on youth of African descent who are integrating into an industrialized society.

**Methods:**

Qualitative data on cardiovascular risk and acclimation to the dominant society were collected from three groups of key informants: (1) community leaders; (2) youth; and (3) a community advisory group.

**Results:**

Youth of Ethiopian descent engaged in the same western diets, computerized social networking, and habits in smoking and alcohol use as did youth from the dominant society. However, informants of Ethiopian descent encountered and witnessed racism, institutional discrimination and evidence of devaluing Ethiopian culture, influencing youths’ ability to integrate into the society.

**Conclusion:**

Immigrant youth of Ethiopian descent face an accumulation of conflicting social support, psychosocial factors, and stressors, including: living in low-income, high-crime areas; encountering pervasive discrimination; acclimating to a new and industrialized culture; and navigating within an often unhospitable society. Contributing to these factors are changes in health behaviors such as adding processed foods and sugary drinks to the diet, increasing heavy alcohol use and substituting screen use for physical activity. The accumulative impact of these factors contributes to the marginalization of youth of Ethiopian descent in the dominant society and perpetuates a cycle of increasing cardiovascular risk.

## Background

Cardiovascular morbidity is linked to many health and social behaviors which are developing in our preteen and teenage years. Yet, among the factors explaining the differences in risk for cardiovascular morbidity in high-income countries are socioeconomic and ethnic/racial minority status [1]. Initially researchers believed that these two variables were virtually the same suggesting that most ethnic/racial minorities were from lower socioeconomic status; however, when studies statistically controlled for socioeconomic status, disparities between the dominant and minority ethnic/racial groups remained [1, 2]. Poverty and minority status contribute to cardiovascular risk, but other factors such as being an immigrant may add further risk. In fact, the mortality rates of some immigrants are higher in their country of origin than in the host country, but these rates may differ based on whether the country of origin was a high-income country or not [3]. Immigrants, arriving to a high-income country from a low income country, encounter a mixture of positive and negative effects that have the potential to both influence their cardiovascular risk and cultural acquisition.

### Theories on cultural acquisition of immigrant and racialization

New immigrants faced with a new culture, including a different language, mores, lifestyle and dress, experience varying levels of embracing the new dominant culture [4]. A term called the “healthy immigrant effect” suggests that recent immigrants possess better overall health, particularly with respect to chronic diseases, compared to native-born residents; however, this “effect” diminishes over time as immigrants engage in unhealthy habits, such as smoking and eating processed foods that are common in high-income country [5, 6]. Immigrant youth, who have arrived from low-income countries are eager to acclimate to their host society, and are particularly vulnerable as they quickly seek to emulate the behaviors of native-born youth and this may include consuming high fat diets with many processed foods and sugary drinks as well as engaging in sedentary activities such as online social networking rather than physical activity [7]. Thus, the effects of cultural acquisition may be accelerated in youth [4, 8].

The six models of cultural acquisition include [9]:

1. Marginalization– the immigrants are neither comfortable in their culture of origin nor in the host culture.
2. Assimilation – the immigrants are completely absorbed into the dominant culture so little evidence of their culture of origin is evident.
3. Acculturation model – the immigrants are completely absorbed into and are competent within the dominant culture, but they *always* will be identified as a part of the minority culture.
4. Alternation model – the immigrants adapt to both cultures by adjusting behavior based depending on the social context and are able to function effectively in both cultures.
5. Multicultural or blended model- the immigrants feel comfortable in both cultures; but do not need to change behaviors.
6. Fusion model – the immigrants’ culture is combined into a melting pot mixing all cultures in the society so neither a minority nor dominant group exists.

Although these categories were designed to represent integration of adults into the host culture, they also have been used for adolescents who are in the developmental stage of formulating their identity [10, 11]. Minority adolescents are not a homogeneous group, and therefore, different adolescents may fit the criteria of different cultural acquisition models [12]. Other characteristics such as socioeconomic status contribute to the youth's self-identity [13] as does perception of the social environment as welcoming or antagonistic [12, 14, 15]. Moreover, in the six models of cultural acquisition described by La Framboise (1993), the model of acculturation, in which the new group *always* will be identified as a minority culture and not part of the dominant society, hints to racialization. Racialization refers to how certain people judge others as not belonging to their group, usually based on any number of characteristics including physical features such as skin color [16, 17].

### Cardiovascular risk and the interplay of cultural acquisition and racialization

Historically, research on cardiovascular risk factors, has focused mainly on health behaviors; however models such as the Healthy Environments Partnership (HEP) are providing a more expansive framework that adds the influences of physical and social environmental to the other cardiovascular risk factors. The section of the HEP model labeled the ‘Proximate Component’ organizes them into the following four elements: (1) health behaviors, (2) social support, (3) stressors, and (4) individual characteristics or psychosocial factor [18]. Health behaviors, consisting of the first element of the HEP model, include diet, exercise, smoking status and habits involving binge or heavy drinking. Social support, the second element of the HEP model, involves family and community participation. Stressors comprise the third element of the HEP model include chronic stress from low socioeconomic status and perceived everyday treatment by society – including racism. Individual characteristics or the psychosocial factor, the fourth HEP model element, is comprised of attitudes and beliefs including feelings such as hopeful/hopelessness and satisfaction/frustration.

Chronic stress is the accumulation of a persistent and continuous exposure to a source of stress such as abuse, severe poverty and racism [19, 20]. Although not completely understood, findings suggest that biological inflammatory processes are produced or exacerbated by chronic stress [21, 22]. Measurement, identification and isolation of the processes and biomarkers reacting to recurring stressors and trauma have yielded mixed results [22]. Nevertheless, large epidemiologic studies have found that after adjusting for many individual characteristics, perceived racism was associated with cardiovascular morbidity such as high blood pressure [23, 24]. Thus, chronic stress may result from racialization (i.e., identified as “others” and faced consistently with exclusionary attitudes and behaviors by the dominant society). In the situation where there is racialization, in which the minority individuals will always be identified as part of the minority and not part of the dominant group, only two cultural acquisition models are possible: marginalization and acculturation.

Chronic stress associated with pervasive racism has been linked to cardiovascular risk in other ways as well. Studies suggest that those coping with racism and exclusion are more likely than others to engage in unhealthy behaviors (i.e., being obese, smoking, and drinking alcohol) [25, 26], and to suffer from hypertension, mental health problems and even early mortality [26]. Accordingly, immigrants who fit the marginalization model, neither feeling comfortable in their culture of origin nor in their host culture, may be more likely to suffer from the higher levels of chronic stress.

### Immigrants: The special case of ethiopian jews in israel

Of the more than 10 % global migrants leaving Africa, 0.6 % are from Ethiopia, one of the poorest countries in the world [27]. One destination of Ethiopian emigrants is Israel. Although Israel is a country populated by many immigrants, refugees, and their descendants (the 2010 Israeli census indicates almost 25 % of the dominant population consisting of Israeli Jews were born outside Israel: 20.0 % from Asia; 25.3 % from Africa; 54.7 % from Europe, America and Oceania [28]), the transfer of Jews from the very rural isolated villages of Ethiopia to Israel was unique. First, they came from a country ranked at the lowest income level in the world to a country ranked among the highest [29]. Second, the Jews of Ethiopia fled from their country due to economic hardships and discrimination, traveling hundreds of miles often on foot to reach planes traveling to Israel [30]. Through airlifts organized in 1984 (Operation Moses) and 1991 (Operation Solomon), thousands of Ethiopian Jews were brought to Israel and placed, as are many immigrants, into absorption centers, a small enclave of buildings where Hebrew language classes, health care, housing and food are provided [30, 31]. Generally, the absorption centers are located in less populated and less desirable areas [32]. Most of the approximately 130,000 Jews of Ethiopian descent in Israel today are either immigrants, or native-born Israelis from those families [33].

The HEP model elements (health behaviors; social support; stressors; and psychosocial factors) *not only* depict cardiovascular risk, *but also* reflect the manifestations of cultural acquisition. We explore these manifestations on youth as described by three groups of Israelis of Ethiopian descent: youth, community leaders, and a group of mothers raising children in Israel.

## Methods

### Study design and participants

This study triangulates three sources of qualitative data to explore the health behaviors, social support, stressors and psychosocial factors influencing cardiovascular health among adolescents. A purposive sample was drawn from three different groups of participants who self-identified as being from the Israeli-Ethiopian community: community leaders (*n* = 4), youth (*n* = 10), and a community advisory group (*n* = 6). We identified and invited for interview community and youth leaders from newspaper articles, internet sites, and non-profit organizations. Community leaders were adults who had been born in Ethiopia, lived in Israel for at least 10 years, and worked with Israeli youth of Ethiopian descent. Youth, ages 18–22, born in Israel or immigrated before age 12, were recruited by referrals, blogs and advertisements. The community advisory group, consisting of mothers who were born in Ethiopia and raising youth ages 15–22, was assembled with the help of a community activist.

### Instrumentation, procedure and interviews

Community leaders and youth were interviewed using in-depth semi-structured, audiotaped interviews with an interview guide containing the specific content areas (i.e., diet, exercise, smoking and alcohol, stressors) to ensure all major areas of the phenomenon were covered but also that participants described cardiovascular health in their own way. These audiotaped interviews were transcribed verbatim. Data from the community group of mothers were collected using a focus group. The focus group was not audio-taped; instead, one facilitator led the group and another facilitator took comprehensive notes. The decision to not audiotape the community group of mothers was decided based on the guidance of the community activist who stated that audio-taping would impede open discussion due to the sensitivity of some topics. All youth, community leaders and community group advisors signed informed consent as approved from the University of Haifa Ethics Committee (# 054–12); all participants received a gift card for their time. Names and identifying details from the interview transcriptions were removed from the participants’ quotes in this manuscript.

### Data analyses

To examine the data, we used thematic content analysis, a systematic method of condensing text into content categories based on explicit rules of coding. Content analysis is a useful method when existing theory or research literature on the phenomenon under study is limited, thus understanding of information from study participants is possible without imposing preconceived theoretical perspectives on the data [34]. We performed the analysis in two main stages. First, we read each transcript separately, identifying initial themes to emerge inductively. In this stage, interviews were read repeatedly to become familiar with the participants’ stories and to identify central issues in each of the separate interviews [35]. These first impressions, thoughts and initial analysis were recorded. In the second stage, we examined themes that had arisen in the initial analysis of each separate interview, seeking connections, similarities and differences among them. Consequently, we identified central themes that emerged from interviews, which characterized and represented the phenomenon under study [36].

## Results

From the interviews focusing on cardiovascular risks, three dominant themes emerged indicating the combined struggle for cultural acquisition to Israeli society and the antagonism that youth encountered: (1) Being like other Israeli youth; (2) Between Ethiopian and Israeli Lifestyle, and (3) Living and Integrating in an Alienating Society

### (1) Being like other israeli youth

The youth demonstrated preference and acculturation to the Israeli lifestyle. They mentioned several attitudes and behaviors indicative of Israeli society. Screen use (i.e., television and computer use including social networking) was high overall (80 %). Internet use was mentioned much more frequently than team sports or exercise.

*When I was in high school, I was hooked on the computer, TV, all, all these things…I would be on the computer four, five hours. (Youth, M: File 8, p.20: 12–14)*

Youths’ goals focused on successful with social integration. They systematically assessed options that would help them succeed in society. They sought self-improvement, and described ways in which they coped with the societal challenges to gain work and continue education.

*I registered into a course…one that should improve your achievements, it’s aimed to encourage Ethiopian youth to reach out for things, to aim for higher roles, to do their best.)Youth, F: File 3, p.11, 13–19)*

Participation in the mandatory military (or community) service also was mentioned as a goal. It is important to note, that although service is mandatory, youth who are considered unsuitable, such as those with criminal records or psychological problems, are excused from duty. Military service represents being a part of Israeli society, a rite of passage. For men, this three-year service begins at age 18 or after completion of high school. Afterwards service is a topic at job interviews and may lead to other opportunities in Israel. Consequently, it not only symbolizes being Israeli, it also influences future societal integration. Stress connected to military service is present for all Israeli youth, but may be even greater for youth of Ethiopian descent, as their fathers, immigrants to Israel, do not have experience with Israeli service and so were unable to provide their sons with information to prepare them for the process.

*What are the most meaningful things in my life?. . . good career is the most important factor. It’s important for me to invest in things related to life, the environment and society where I live. I always try to give, if I can. . . I have no [educational support or] background from home. So I've gathered information from school and from people who came to explain to us about the military service. [From them]…I’ve figured out how to act and what military service is like . . . [and it is the main factor that has motivated me] to become integrated in Israeli society. I want to contribute to my society and I’m also interested in having a military career. I immigrated at age 11. I have a different perspective than other youth my age. At school I realized that I couldn't get what I needed – whether it’s language or acclimating into Israeli society. So, I decided to go to a boarding school. .there it would be easier for me socially, and probably - also easier for my studies. (Youth, M: File 10, p.3 3–9)*

Youth of Ethiopian descent described the stress of trying to make parents proud. Since Ethiopian society is patriarchal, the father’s opinion is important. Culturally-based expectations are a part of youth’s thoughts and beliefs, and as such, influence behavior.

*When I went into the army, it was really tough for me. I almost broke The sole reason that I wanted to remain there is my parents. …also I say to myself that I'm not allowed to quit on myself, especially for my father. (Youth, M: File 8, p.4, 7–8)*

### (2) Between Ethiopian and Israeli lifestyle

Youth participants indicated knowledge of good health habits including a diet with vegetables, fruits, low amounts of fried food, and regular exercise. While all youth reported eating fast foods (100 %), several spoke about the dangers of processed foods, and clearly indicated that Ethiopian foods and lifestyle were much healthier than Israeli foods and lifestyle.

*I think that …the food here in Israel is synthetic. It’s industrialized food compared to the food in Ethiopia (Youth, F: File 1, p14, 8–10)*

*in Ethiopia, the lifestyle was active in that there was physical work, agricultural work [and] there were no processed foods. The food there was natural. They [Ethiopian] ate the food that they grew themselves (Leader: File 1, p2-3, 25–27, 1–5)*

Yet, virtually all identified fast, processed foods such as fried chicken breast, hamburgers, pasta, or French fries as their foods of choice. Cola drinks also were mentioned frequently. Most youth indicated that they did not eat with their families during the week days, grabbing whatever food was available, but the Sabbath meal was an exception when the entire family ate together. The Sabbath meal among many Jews is a family gathering; and it was at this meal that several youth and community leaders mentioned that Injera (Ethiopian bread) was served.

Community advisors and leaders (100 %) agreed that youth preferred non-traditional foods, but reported that this preference also may indicate the belief that traditional, Ethiopian culture was less valued in the dominant Israeli society.

*Ethiopian youth are influenced by everything…cigarettes, alcohol, all that, [although the other Israeli youth] are more addicted to it. (Youth, M: File 5, p.16: 5–8)*

The parent–child relationships of urban Israel and rural Ethiopia also clashed. In Ethiopia, parents’ wishes were obeyed, while in Israeli society, many youth debate and question parents’ requests. Youth of Ethiopian descent were caught in the middle and pulled by these two very different lifestyles. One example was:

*In Ethiopia, whether the father or mother would say something, you would not look him in the eye, and you would obey their commands. As if God spoke to you, you would do it right away. But now, say, the child refuses…the parents think it’s sass and lack of education. In short, it’s a mess, they [the youth] do not understand them [the parents] and they [the parents] do not understand you and that creates problems and miscommunication (Youth, F: File 3, p14-15: 23–27, 1–3)*

Being a child to an immigrant family also affected the youths’ resources. Being immigrants themselves, parents were unable to provide the information that would prepare and support their children for the acquisition of a new culture. Youth had the stress of obtaining this information by themselves. At times, parents were supportive and helpful, but at other times parents further added to their children’s stress by requiring their children’s help with understanding the language and culture.

*Yes, parents…they really need to have things explained to them, someone to translate for them…I see that the Israeli [youth], they know Hebrew from home, they speak and learn, but for us for example I talk to [and reassure] my parents so they won’t be stressed (Youth, M: File 6, p.13, 10–17).*

### (3) Living and integrating in an alienating society

Community advisors and leaders (100 %) described the stress that youth experienced trying to succeed in problematic neighborhoods where there were the obstacles of poverty, alcohol, delinquency, and apathy, which were pervasive and seductive, and contributing to the individuals failing in the society. Additionally, the alienation caused by racism was mentioned by all respondents (100 %) – youth, community advisors and community leaders.

Discrimination and racism against Ethiopian immigrants were described as ubiquitous in Israeli society and a part of life that added obstacles and stress, and a barrier to being successful. Many described the event in 1996 when it became public that donated blood from Ethiopian-Israelis was discarded based on the belief that it might be infected with HIV [37]; yet, most youth had either not been born or were less than five years old at the time. The historical memory of racism was augmented by discrimination encountered in everyday life at school, and contributed to the feelings of anger, indignation and humiliation and the stress of calming oneself.

*Do this, do this, do this, and they (make you angry) drive you crazy and you don’t know what you are saying, and then [the counselor] goes and says this to the head teacher, to the social worker, the whole thing, and then you need to go to the director’s office…you get nervous. …she says Ethiopians are like this and like that. (Youth, F: File 4, p7-8: 1–27, 1–4)*

*They [the teachers] don’t give high level instruction, and then accordingly there aren’t high expectations [of the youth] (Leader: File 1, p.18, 1–2)*

Often there was the description of being discouraged, demeaned and held back in addition to having to work more than youth who were not from families of Ethiopian descent.

*[An Ethiopian-Israeli youth] tries to integrate into society and sometimes gets rejected. Every time he makes headway, always there is the stage that he gets grabbed and slapped, and grabbed and slapped, and then again has to return home (Youth, M: File 104, p. 14: 18–20)*

Some youth did not have friends outside the community. They appeared to make a distinction between themselves, as Ethiopian, and not Israeli.

*I don’t know many Ethiopians [youth] who have really good Israeli friends. (Youth, M: File 10, p. 14, 23–24*)

Other youth did not have friends outside the community. They referred to themselves as Ethiopian and the other youth as Israelis.

*I feel, alone…isolated. I think that Israelis and Ethiopians should be better integrated…I connect more to Ethiopians (Youth, M: File 6, p.10, 23–27)*

Some youth also described the stresses of school as well as the societal influences that had a positive or negative impact on their future success in Israeli society.

*In the neighborhood… [there are Ethiopian-Israeli youth who have] no education.. … it’s a kind of pressure, stress…this is a relatively troubled neighborhood, so [you live] here, go to school here, you don’t learn, and so you don’t go to school, and obviously, people here make trouble, and that’s it. And once you start to hang out with them slowly, slowly it’s as if, catches up with you too. (Youth, M: File 9, p.19: 5–9)*

Descriptions of experiences with discrimination were common. Still, there were youth who were coping and resilient. They were dedicated to integrating into society despite the obstacles they encountered.

*But I try to adjust, that’s life here. There are many cases [of racism]. With Ethiopians, it’s known there’s racism … Not everyone, I don’t say that it’s everyone, but there are lots of cases, there’s no shortage of cases. (Youth, F: File 3, p. 22, 16–19)*

*Today I am more Israeli than Ethiopian. I am busy with what I have here, not with the past. I try to move forward, even though there is racism, and there is discrimination. Still, I am moving [towards integrating] into the Israeli society rather than Ethiopian society. …I do not give up. I came here for one reason, one goal: to become part of this nation. (Youth, M: File 5, p2-3: 21–27,1)*

Friends served as confidants and supporters, but sometimes, also encouraged smoking and heavy drinking. Youth described how their neighborhoods were infested with crime and so there was the constant seduction of alcohol, drugs and criminal activity. Again, youth described the chronic stress and struggle of being seduced by the marginalized culture of crime and poverty that surrounded them and the desire to succeed in society.

*Being here [in this neighborhood] without getting involved in all the nonsense [including not drinking] alcohol…that helped me to get to where I am today. (Youth, M: File 5, p.22: 11–17)*

*[Friends] are influential… they do all sort of things. Crime. Then I don’t join in all that, it’s not right for me. I don’t want to get involved in that and to end my life at an early age.)Youth, M: File 7, p.25-27, 1–4)*

*The environment influences [the ability to succeed] because newcomers were brought into these depressed neighborhoods, and depressed neighborhoods invite all sorts of problems…. (Leader: File 2, p12, 3–4)*

Several youth and leaders voiced sorrow and regret that the language, food and other habits from Ethiopia were being replaced by those of Israel. They appeared to be torn between conflicting beliefs – adopting Hebrew was necessary to integrate into Israeli culture; however, Hebrew and the non-traditional Israeli culture seemed to be replacing, rather than adding to Ethiopian culture. This too added stress.

*[Some are adapting to the Israeli culture, but] on the other hand, [they have lost many of our customs and do not even] eat Injera. (Leader: File 4, p. 37, 20–26)*

*It’s not just a political thing, I also asked my mother and think that in order for us to be well assimilated, they [the parents] sort of had to speak Hebrew with us. (Leader: File 4, p. 44, 20–22)*

*No, really, the [skin] color is the only thing that will remain [of our culture]. I say that I’m Ethiopian. Okay. But can I cook the food? Do I dress traditionally? [The language that] I have at the tip of my tongue is Hebrew. (Leader: File 4, p. 45, 10–13)*

## Discussion

All four elements of the HEP model’s proximate component, associated with increased cardiovascular risk, were described as being present in youth of Ethiopian descent including risky health behaviors (i.e., diets of fast foods, high screen use versus less exercise, heavy alcohol use), lack of social support (i.e., obstacles to social participation and integration), stressors (i.e., living in poor neighborhoods, racism), and problematic psychosocial factors (i.e., frustration, hopelessness). With the exception of the stressors, these factors reflect the efforts to acclimate to Israeli society.

The role of the stressors such as racism was ubiquitous in youth of Ethiopian descent. Racism interferes with societal integration while contributing to frustration, disappointment and anger [38]. Yet, even without the continued stress of racism, socioeconomic factors demonstrated significant correlations to poor health, morbidity and mortality in refugees, migrants and immigrants [39, 40]. However, when pervasive racism in the dominant society is added to the existing accumulation of factors, integration into Israeli society is daunting, and feelings alienation and marginalization increase. This two-way process, of being pushed away by dominant society and feeling at a disadvantage reflects the cycle of marginalization. It is a reciprocal process, whereby newcomers (immigrants) exhibit characteristics different from the greater society, and in response, the individuals from the greater society indicate their unwillingness to bestow membership to these newcomers [4]. This reciprocal process was evident as youth of Ethiopian descent who referred to themselves as Ethiopian and to other youth, who were not of Ethiopian descent, as Israelis. Marginalization has been reported in other industrialized cultures in which individuals of an ethnic/minority group are surrounded by discrimination and consistently receiving direct and indirect messages that they are not part of the dominant community [41, 42].

Some studies suggest that cardiovascular morbidity in adult immigrants of African descent is higher in societies where the dominant population is western, urban and ethnically white [43, 44]. One possibility for this finding is the accumulation of chronic stressors resulting from discrimination and the consistent societal behaviors contributing to marginalization. Historical events may further contribute to these chronic stressors. Such “historical” memories included an event in Israeli history when blood donated by Israeli Jews of Ethiopian descent was discarded due to the belief that it was infected with the AIDS virus [37]. Historical and collective memories of trauma contribute to feelings of marginalization. Ironically, the same collective or historical memories that contribute to feelings of marginalization, also contribute to forming our identities and connection to a particular social, political or ethnic group [45, 46].

Increased cardiovascular risk also results from lifestyle changes characteristic of more industrialized culture, such as eating unhealthy foods, recreational use of the internet rather than engaging in exercise, and smoking. Cardiovascular morbidity including increasing rates of diabetes have been reported among African immigrants; and the explanation suggested for this increase is that after many generations of consuming foods that were low in fat and sugar, the African immigrant metabolism has difficulty adjusting to the western diets that are high in fat and sugar [47, 48]. Although several youth mentioned with pride that they, as well as many other Israeli youth of Ethiopian descent, excelled in running and team sports, many more reported use of the internet rather than engaging in exercise. This too is consistent with other Israeli youth who tend to spend long hours on the internet [49]. Still, youth who engaged in these more Israeli but less healthy behaviors, which contributed to cardiovascular risk, also were exhibiting indications of acculturation.

Binge or heavy drinking is another lifestyle choice of youth in many industrialized cultures. Many youth of Ethiopian descent admitted to drinking, much fewer reported smoking. This description is consistent with current trends in Israel, where although the rate of smoking is decreasing, binge drinking and heavy alcohol consumption are increasingly problematic [50]. Still, the health behaviors described by youth of Ethiopian descent were no different than those documented of the general Israeli population of youth. Drinking alcohol and smoking also were found in Ethiopia; but youth, the community leaders and the community advisors noted that such habits were rare in Ethiopia. Youth were aware that that smoking and drinking were both unhealthy and problematic, and often lead to negative consequences. However, these negative behaviors were viewed as being like other Israeli youth; and so youth who desired to blend in, adopted these negative behaviors which paradoxically pushed them further towards marginalization. Immigrant youth and youth born to immigrant parents may be more vulnerable to adopting negative risky behaviors that are exhibited by the youth of the dominant culture.

**Fig. 1:**
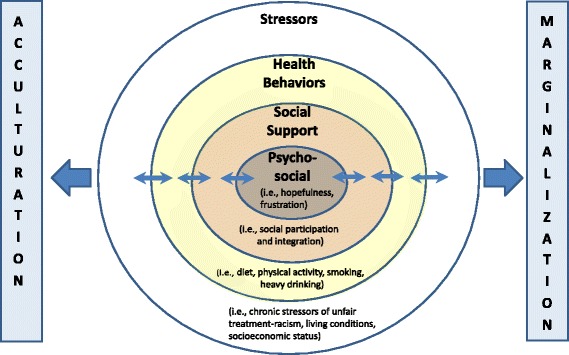
Cardiovascular risk factors and cultural acquisition strategies for immigrant youth of African descent

Findings suggest that their acclimation to the dominant Israeli society may be associated with the four cardiovascular risk factors found in the proximate component of the HEP model (i.e., health behaviors; social support; stressors; and characteristics or psychosocial factors), and influenced by the two types of cultural acquisition common among immigrants with different physical characteristics (i.e., marginalization and acculturation) (See Fig. 1). Resilient youth, those who were hopeful and envisioned a positive future despite the obstacles and stressors [51], demonstrated more characteristics of acculturation. Moreover, the effects of being hopeful propelled them to find work or study as a means of thriving and acculturating into Israeli society. Their ability to cope and the desire to succeed led these youth to make lifestyle choices that initiated a self-perpetuating cycle, which contributed to less stress, better health habits and increased acculturation into Israeli society. Other youth were overwhelmed with the emotions of anger and frustration from discrimination, poverty and the rejection from the dominant society. They were more likely to engage in heavy alcohol consumption drinking and smoking (which has been problematic in Israeli youth [50]), paradoxically believed that adopting these negative behaviors would help them blend in and acculturate to Israeli society. However, such characteristics contribute to marginalization.

This study contributes to existing literature by illustrating that the stressors of racism and discrimination are pervasive for youth of African descent and influence the connection between cardiovascular risk behaviors and cultural acquisition to an industrialized society. However, this study has limitations. Although results were meant to reflect immigrant youth of Ethiopian descent in Israel, the purposive samples of Ethiopian-Israeli respondents were small and enrolled from northern Israel, which may reduce the external validity to other Israeli-Ethiopians. Moreover, findings must be generalized with caution to other African countries’ immigrants, particularly as the Ethiopian-Israeli youth of African descent in Israel are comprised primarily of the Jewish minority of Ethiopia. Moreover, the Israeli policies, benefits and procedures designed to assist new immigrants are unique and may not be similar to those of other countries. Still, the study employed samples representing three perspectives (community leaders, youth and advisory group), and all samples were comprised of individuals of Ethiopian descent.

## Conclusion

Several high-income countries like Israel boast about their heritage as immigrant countries, but their strategies to assist in the societal integration of immigrants, particular those of color who emigrate from developing, less industrialized countries, may be lacking; and this may contribute to increases in chronic stress in its immigrant youth. Several studies already have found an association between perceived racism and adulthood cardiovascular morbidity [23, 24]. Based on our findings, immigrant youth of color experience pervasive racism and these perceptions contribute to chronic stressors and other factors connected to cardiovascular risk. In an increasingly heterogeneous world, it is important for societies to identify and implement societal strategies that improve their abilities to help immigrant youth with different physical appearance (i.e., skin color) to integrate into the host culture. While some suggest that a multicultural society is the goal [4], that would not be possible in a society where, due to discrimination, immigrant groups feel the impetus to abandon their own culture and replace it with the host country’s culture. Equally importantly, the dietary, exercise, heavy alcohol consumption and smoking habits along with the stressors acquired due to the societal acclimation have public health implications on the cardiovascular risk among immigrant youth or first-generation descendants who are of color and from low-income country.

### Epilogue

Beginning in May 2015, approximately a year and a half after this study’s interviews and focus group were completed, several demonstrations and protests erupted in cities all over Israel describing the frustration that Ethiopian-Israelis experienced with the racism that they experienced in education, employment, and everyday life. Newspaper quotes from Ethiopian-Israelis reflected sentiments of being ostracized and unwelcome in Israeli society in the same way that quotes from youth, community leaders and the advisory group did in this study:

“*I do not need a hug, your patronizing attitude*” [52].

*“We are Israelis just like everyone else…”* [53].

Ethiopian-Israelis comprise less than 5 % of the population, yet the demonstrations continued for at least a month throughout the country. Such persistence reflects the high levels of stress, and the connection between the public context, public policy and public health.
